# Clinical Features and Inflammatory Markers in Autoimmune Encephalitis Associated With Antibodies Against Neuronal Surface in Brazilian Patients

**DOI:** 10.3389/fneur.2019.00472

**Published:** 2019-05-14

**Authors:** Paulo Ribeiro Nóbrega, Milena Sales Pitombeira, Lucas Silvestre Mendes, Mariana Braatz Krueger, Carolina Figueiredo Santos, Norma Martins de Menezes Morais, Mateus Mistieri Simabukuro, Fernanda Martins Maia, Pedro Braga-Neto

**Affiliations:** ^1^Division of Neurology, Department of Clinical Medicine, Universidade Federal do Ceara, Fortaleza, Brazil; ^2^Neurology Service, Hospital Geral de Fortaleza, Fortaleza, Brazil; ^3^Department of Neurology, Faculdade de Medicina, Hospital das Clinicas HCFMUSP, Universidade de São Paulo, São Paulo, Brazil; ^4^Unichristus Medical School, Unichristus, Fortaleza, Brazil; ^5^Child Neurology Service, Hospital Infantil Albert Sabin, Fortaleza, Brazil; ^6^Medical Sciences Post-Graduation Program, Universidade de Fortaleza, Fortaleza, Brazil; ^7^Center of Health Sciences, Universidade Estadual do Ceara, Fortaleza, Brazil

**Keywords:** autoimmune encephalitis, Inflammatory biomarkers, neuronal surface antibody, NMDAR, LGI1, AMPAR, low-income population

## Abstract

Acute encephalitis is a debilitating neurological disorder associated with brain inflammation and rapidly progressive encephalopathy. Autoimmune encephalitis (AE) is increasingly recognized as one of the most frequent causes of encephalitis, however signs of inflammation are not always present at the onset which may delay the diagnosis. We retrospectively assessed patients with AE associated with antibodies against neuronal surface diagnosed in reference centers in Northeast of Brazil between 2014 to 2017. CNS inflammatory markers were defined as altered CSF (pleocytosis >5 cells/mm^3^) and/or any brain parenchymal MRI signal abnormality. Thirteen patients were evaluated, anti-NMDAR was the most common antibody found (10/13, 77%), followed by anti-LGI1 (2/13, 15%), and anti-AMPAR (1/13, 7%). Median time to diagnosis was 4 months (range 2–9 months). Among these 13 patients, 6 (46.1%) had inflammatory markers and when compared to those who did not present signs of inflammation, there were no significant differences regarding the age of onset, time to diagnosis and modified Rankin scale score at the last visit. Most of the patients presented partial or complete response to immunotherapy during follow-up. Our findings suggest that the presence of inflammatory markers may not correlate with clinical presentation or prognosis in patients with AE associated with antibodies against neuronal surface. Neurologists should be aware to recognize clinical features of AE and promptly request antibody testing even without evidence of inflammation in CSF or MRI studies.

## Introduction

Autoimmune encephalitis (AE) is increasingly recognized as one of the most frequent causes of encephalitis (1, 2). The identification of antibodies against neuronal surface antigens as biomarkers of treatable neurological syndromes has changed the approach to encephalitis and other inflammatory central nervous system (CNS) disorders (1). The differential diagnosis of AE may be complicated because signs of inflammation on neuroimaging or cerebrospinal fluid (CSF) studies may be absent, especially in patients over 60 years of age (3). This raises the question of whether these findings would be replicated in consecutive, unselected patients without age restriction. In Brazil there is a small series of three patients reported with anti-NMDAR antibodies in the city of Brasília (4), and a study in rapidly progressive dementia in which 10 cases of anti-NMDAR encephalitis were reported in São Paulo (5). The aim of this study was to characterize clinical features and outcome in a cohort of consecutive patients with AE from a single city in Northeast Brazil and to compare patients that presented or not findings suggestive of active inflammation.

## Materials and Methods

### Patients and Clinical Data

We retrospectively identified consecutive patients whose serum or CSF samples tested positive for neuronal antibodies in the city of Fortaleza, state of Ceará, northeast Brazil, from January 2014 to March 2017. This study was conducted in the main neurological comprehensive care centers of our city (Hospital Geral de Fortaleza, Walter Cantidio University Hospital and Albert Sabin Children's Hospital), covering a population of almost 2.7 million. Thus, we believe that most patients diagnosed with AE during this period were included in our cohort.

Blood and CSF samples analyses were performed at IDIBAPS, Barcelona, with support from Dr. Josep Dalmau. All samples were screened for reactivity using rat brain sections and then through cell-based assay with HEK293 recombinantly expressing N-methyl-D-aspartate receptor (NMDAR), leucine-rich glioma inactivated-1 (LGI1), contactin-associated protein-like 2 (CASPR2), α-amino-3-hydroxy-5-methyl-4-isoxazolepropionic acid receptor (AMPAR), gamma-aminobutyric acid (GABA)b receptor or GABAa receptor as previously reported (6).

AE was defined by the presence of all three of the following criteria: (1) subacute onset (rapid progression <3 months) of working memory deficits (short-term memory loss), altered mental status, or psychiatric symptoms; (2) at least one of the following: new focal CNS findings, seizures not explained by a previously known seizure disorder, CSF pleocytosis (white blood cell count > 5 cells/mm^3^), or magnetic resonance imaging (MRI) features suggestive of encephalitis; and (3) reasonable exclusion of alternative causes (1). Exclusion criteria included syndromes predominantly involving the spinal cord or peripheral nerve.

Electroencephalogram (EEG) monitoring was performed in all patients. The following EEG variables were collected: presence or absence of electrographic seizures; clinical or subclinical seizures; diffuse slowing; focal slowing; rhythmic delta activity; excessive beta activity; and presence of extreme delta brush. MRI included T1-weighted, T2-weighted, fluid attenuated inversion recovery (FLAIR), diffusion weighted imaging (DWI) and gradient echo sequences. CNS inflammatory markers were defined as altered CSF (pleocytosis > 5 cells/mm^3^) and/or any brain MRI abnormality suggestive of encephalitis (mesial temporal T2 hypersignal or lesions suggestive of demyelination).

The modified Rankin Scale (mRS) was used to assess disability at onset, at the worst clinical status and at the last visit. A good outcome was defined as mRS score of 0–2 at the last follow-up and a poor outcome as mRS score > 2. Tumors were screened for at the initial presentation and every 6 months with thoracic and abdominal computed tomography and vaginal ultrasound or ovarian MRI for ovarian teratomas.

### Statistical Analyses

Categorical variables were described as absolute frequencies and percentages, while numerical variables were described as medians and ranges. Comparative analyses between patients with and without inflammatory changes were performed using the Mann-Whitney *U* test or 2-sample *t*-test as appropriate for continuous variables, and Fisher's exact test for categorical variables. Two-tailed *p*-values of < 0.05 were considered statistically significant. Statistical analyses were performed IBM-SPSS version 18.0 (IBM, Armonk, NY, USA).

### Ethical Approval

All patients gave their written consent for the storage and use of clinical samples for research purposes and to be included in this report. The study was approved by the local ethics committee (number of approval: 2.652.778).

## Results

### Demographic and Clinical Features

Among 13 patients identified, 9 (69%) were female and median age was 17 years (range 4–75 years). Anti-NMDAR was the most common antibody found (10/13, 77%), followed by anti-LGI1 (2/13, 15%), and anti-AMPAR (1/13, 7%). Median time to diagnosis was 4 months (range 2–9 months) Before an AE was considered, patients received other diagnosis including herpes simplex encephalitis, primary psychosis, and neuroleptic malignant syndrome.

The most common initial presentation was encephalopathy with memory loss and behavioral changes suggestive of AE seen in 11/13 (84%): all 10 patients with anti-NMDAR and 1 patient with anti-AMPAR. Psychiatric symptoms presented before neurologic dysfunction in 4/13 patients. Dyskinesias, typically orofacial, were present in 9/13 patients and hand dyskinesias in 5/13. Dystonia was seen in 3/13 patients. Faciobrachial dystonic seizures (FBDS) followed by encephalopathy and memory loss were present in both anti-LGI1 patients. Only one patient with anti-NMDAR presented with refractory status epilepticus followed by orofacial and hand dyskinesias, dystonia and encephalopathy after resolution. Hyponatremia was present in three patients, one with anti-AMPAR and in both with anti-LGI1, and improved after immunotherapy. Regarding associated tumors, there was only one case of small cell lung carcinoma in the patient with anti-AMPAR. Table 1 presents clinical features of patients with AE.

**Table 1:** Summary of clinical presentations, paraclinical investigations and follow-up data of patients with autoimmune encephalopathy.

**Case number**	**Age**	**Antibody**	**Clinical presentation**	**CSF**	**MRI**	**EEG**	**Time to diagnosis** **(months)**	**mRS** **(diagnosis)**	**mRS** **(last visit)**
1	16	NMDA	Psychosis, seizures, encephalopathy, rigidity, dyskinesias, dysautonomia	Cells-2, Protein-57	Occipital subcortical hyperintensities	Extreme delta brush	4	5	0
2	16	NMDA	Seizures, encephalopathy, dyskinesias, dysautonomia, akinetic mutism	NL	NL	Extreme delta brush	4	5	0
3	17	NMDA	Psychosis, seizures, encephalopathy, myoclonus, dyskinesias, dysautonomia	NL	NL	Slow theta and delta waves	4	5	0
4	26	NMDA	Encephalopathy, dyskinesias, dysautonomia, dystonia	Cells-336, Protein-16	Cortical hyperintensities	Diffuse slowing	2	4	2
5	26	NMDA	Psychosis, myoclonus, encephalopathy, dyskinesias, seizures, dyasutonomia	Cells-22, Protein-20	NL	Diffuse slowing	4	4	0
6	4	NMDA	Seizures, agressiveness, encephalopathy, dyskinesias, akinetic mutism	NL	NL	Slow activity	3	5	4
7	5	NMDA	Encephalopathy, dyskinesias, dystonia	NL	NL	Diffuse slowing	4	4	0
8	14	NMDA	Status epilepticus, Encephalopathy, dyskinesias, dystonia	NL	NL	Status Epilepticus	5	5	6
9	23	NMDA	Psychosis, akinetic mutism, dyskinesias, dysautonomia	Cells−197, Protein-21	NL	Diffuse slowing	3	5	0
10	75	LGI1	Faciobrachial dystonic seizures, encephalopathy, memory loss, hyponatremia	Cells−10, Protein-40	Right basal nuclei and hipocampal	Diffuse slowing	6	5	3
11	73	LGI1	Faciobrachial dystonic seizures, encephalopathy, memory loss, hyponatremia	NL	NL	Diffuse slowing	9	4	0
12	67	AMPA	Hyponatremia, agitation, insomnia, memory loss	NL	NL	Diffuse slowing	3	4	3
13	17	NMDA	Seizures, agressiveness, mutism, memory loss, encephalopathy	Cells-16, Protein-29	NL	Diffuse slowing	5	5	1

*NMDA, N-methyl-D-asparthate; LGI1, Leucine-rich-glioma-inactivated-1; AMPA, α-amino-3-hydroxy-5-methyl-4-isoxazolepropionic acid; NL, Normal; CSF, Cerebrospinal fluid; MRI, Magnetic resonance image; mRS, modified Rankin Scale*.

### Treatment and Outcomes

Treatment consisted in intravenous methylprednisolone alone (3/13, 23%), methylprednisolone with immunoglobulin (9/13, 69%), or immunoglobulin alone (1/13, 7%). Most patients (8/13, 61%) responded to the first-line therapy. In three patients with no significant improvement after 2 weeks, a second-line therapy was done with rituximab, and in one patient combined with cyclophosphamide. Patients were severely impaired at initial presentation, with a median mRS score of 5 (range 4–5). Most showed significant improvement after treatment, where 9/13 (69%) achieved a good outcome (mRS ≤ 2). Median follow up duration was 22 months (range 3–48). Three patients had a mRS ≥ 3 at the last visit, including one patient with anti-NMDAR who died of central line associated sepsis while receiving parenteral nutrition for a drug-induced pancreatitis (immunoglobulin had been administered 2 weeks earlier with neurological improvement). Maintenance treatment was conducted at the decision of attending physicians. Most patients received oral steroids for a variable period (median of 5 months). None received chronic immunosuppression. There were no relapses during follow-up.

### CNS Inflammatory Markers and EEG Findings

Most patients had normal MRI (10/13, 77%). From those with MRI abnormalities (3/13, 23%): two (both with anti-NMDAR) had extralimbic findings with nonspecific cortical hyperintensities; and one had bilateral hippocampal hyperintensities compatible with limbic encephalitis and left basal ganglia hypersignal (a case of anti-LGI1 with unilateral FBDS). Follow up MRI scans were performed in two patients. In one of the anti-LGI1 patients atrophy of right caudate and putamen was observed after 1 year. In an anti-NMDAR patient complete resolution of subcortical abnormalities and no atrophy were observed after 8 months. CSF pleocytosis was seen in less than a half of patients (5/13, 38.4%). None of the patients had positive oligoclonal bands. All 13 patients showed EEG abnormalities; they were mostly nonspecific slowing of baseline activity (10/13, 77%). Two patients, both with anti-NMDAR, showed an “extreme delta brush” pattern. One patient with anti-NMDAR presented with status epilepticus. In both patients with FBDS, there was no EEG correlation with abnormal movements.

Seven patients (53.8%) had no signs of inflammation on CSF or MRI. No evidence of inflammation was seen in half of patients with anti-NMDAR, in one patient with anti-LGI1 and in the only patient with anti-AMPAR. A good outcome at the last visit was seen in 83.3% of the patients with inflammatory changes and in 57.1% of those without these changes (*p* = 0.559). No significant differences in age of onset, response to immunotherapy, time to diagnosis and prognosis were observed between patients with or without inflammatory markers. Table 2 shows a comparison of clinical features between patients with AE with and without inflammatory changes.

**Table 2:** Comparison between patients with autoimmune encephalitis with and without markers of inflammation in brain MRI and CSF.

**Variables**	**Without inflammation** **(*n* = 7)**	**With inflammation** **(*n* = 6)**	***P***
Age, median (range)	16 (4–73)	24.5 (16–75)	0.197
Female, *n* (%)	85.7%	50%	0.265
Diagnostic delay, median, months	4.0	3.5	0.371
**Clincal Syndrome At Presentation**			
Possible encephalitis^a^	6 (85.7%)	5 (83.3%)	0.906
FBDS	1 (14.3%)	1 (16.7%)	0.906
Hyponatremia, *n* (%)	2 (28.6%)	1 (16.7%)	0.612
mRS at last visit, median	0	0.5	0.641
**Status At Last Visit**			
mRS 0–2, *n* (%)	4 (57.1%)	5 (83.3%)	0.559
mRS 3-6, *n* (%)	3 (28.6%)	1 (16.7%)	

*FBDS, faciobrachial dystonic seizures; mRS, modified Rankin score*.

a *According to the criteria of Graus et al. (1)*.

## Discussion

To the best of our knowledge, this is the largest cohort of patients with AE reported in Brazil. The estimated incidence rate was 0.16/100,000 person-years, five times lower than the incidence of 0.8/100,000 found in a study in Minesotta (7). We found a high frequency of normal MRI and CSF studies and our findings suggest no correlation between inflammatory markers and clinical presentation or prognosis. Some studies have found evidence of association of CSF changes with a worse outcome (8, 9) and MRI changes, particularly cerebellar atrophy in follow-up MRI (10), was negatively correlated with outcome. However, a recent systematic review suggested that early CSF and MRI abnormalities did not demonstrate a strong relationship with patient outcomes (11).

Major diagnostic criteria of encephalitis (of any cause) include the presentation of decreased level of consciousness, neuroimaging findings suggestive of inflammation, and CSF pleocytosis (12). Yet, a recent study applied the newly proposed AE diagnostic criteria to patients over 60 years of age. The authors confirmed these criteria are appropriate to identify patients with possible AE in the absence of evidence of CNS inflammation (3).

The most common antibody found in our study was anti-NMDAR, as reported in other series of AE patients (13). A recent study of 29 cases of anti-NMDAR encephalitis reported an absence of inflammatory markers in CSF in almost 50% of patients, and a delayed time to diagnosis in these cases (14). In our study, time to diagnosis was similar in anti-NMDAR encephalitis patients regardless of their inflammatory status.

NMDAR antibodies bind to an extracellular conformational epitope region close to the amino acid 369 of the GluN1 NMDAR subunit and reduce the receptor density leading to a reversible direct neuronal dysfunction (6, 15). Other autoantibodies may act through different mechanisms. GABAb receptor antibodies relocate the receptor to extrasynaptic sites; AMPAR antibodies reduce the receptor density at synaptic and extrasynaptic sites along with a reduction in miniature excitatory postsynaptic potentials; and LGI1-antibodies block the binding of LGI1 to ADAM22, resulting in a decrease of AMPAR (15–18). The pathogenesis of AE is probably more related to direct neuronal dysfunction caused by these antibodies than to inflammatory infiltrates or blood-brain barrier abnormalities (19). This could possibly explain why there are no signs of CNS inflammation on CSF or MRI in many cases. In both LGI1- and CASPR2-associated CNS syndromes, these antibodies are of IgG4 subclass, which do not fix complement, and the CSF is normal in most patients (20, 21).

Possible triggers for autoimmune response are under investigation. Ectopic expression of NMDAR in ovarian teratomas, for instance, is thought to trigger autoimmune response in paraneoplastic anti-NMDAR encephalitis (22). Recent literature strengthen the hypothesis of an infectious trigger based on the presence of a viral prodrome in the majority of non-paraneoplastic anti-NMDAR encephalitis, the development of NMDAR antibodies in 20% of patients after herpes simplex encephalitis and detection of past non-encephalitic herpes simplex virus 1 (HSV-1) infection in nearly half of the patients with anti-NMDAR encephalitis vs. less than a quarter of the controls (23, 24). Studies in animal models also demonstrate that exposure to HSV-1 can elicit production of NMDAR antibodies as well as reduction of expression of these receptors in the hippocampus of mice exposed to serum of patients with anti-NMDAR encephalitis (25). Some cases of anti-NMDAR encephalitis following mycoplasma, Epstein–Barr, varicella zoster, or influenza infections have also been reported (26–28). Molecular mimicry and dysregulation of immunoregulatory pathways are some of the mechanisms proposed for the link between infections and induction of CNS autoimmunity (16). It is possible that other infectious agents, including zika and chikungunya viruses, which had high incidence rates in Northeast Brazil during the period of this study, might also act as activators of CNS immune response.

Concerning anti-LGI1 encephalitis, we observed a good response to initial treatment in the patient with normal MRI, while the one with T2 hyperintensities in putamen and caudate had a poorer response to first-line therapy with only slight improvement of FBDS and required additional therapy with rituximab. It has been suggested that these signal abnormalities are more common in the presence of FBDS and are related to time since disease onset, with T2 hypersignal disappearing with disease progression (29). Whether this could be related to disease severity is still not clear (30, 31).

In our cohort, only two patients had follow-up imaging for analysis. In both cases, associated with anti-LGI1 and anti-NMDAR, no hippocampal atrophy was observed as previously reported (32). In the follow-up image of the anti-LGI1 encephalitis, caudate and putamen atrophy was observed, and this patient presented a higher frequency and duration of FBDS. This has also been noticed in other series and raises the question about the nature of these events, suggesting that involvement of the basal nuclei causes the stereotyped movements and that they are ultimately a movement disorder rather than a seizure (32, 33).

The importance of testing new onset refractory status epilepticus (NORSE) patients for neuronal surface antibodies has been previously discussed, as many of these cases might have an autoimmune etiology (34). In our cohort, one patient with intellectual disability and stable epilepsy deteriorated and developed prolonged seizures progressing to status epilepticus, behavior changes, and hand dyskinesias (piano playing) and was then diagnosed as anti-NMDAR encephalitis. Testing patients with severe worsening of a previous epilepsy disorder without an obvious cause for deterioration may be warranted in some settings.

The lack of difference between patients with and without inflammatory changes in our study might be due to the small sample size, heterogeneity and lack of representativeness, since cases with only three antibodies types were included with most of the cases being anti-NMDAR. However, we believe this cohort is closer to a “real-world” scenario, as our centers are not specialized in autoimmune encephalopathies and we analyzed all patients who tested positive for neuronal antibodies.

The present study was the first to provide an estimate of incidence of AE in a developing country, where access to MRI and EEG is very limited and antibody testing is not available commercially. Our findings reinforce the importance of previously reported diagnostic clues like FBDS and a syndrome suggestive of anti-NMDAR encephalitis for diagnosis, as well as the sensitivity of Graus criteria for possible autoimmune encephalitis, allowing for early initiation of immunotherapy before antibody results are available. We also report on a good outcome despite many challenges to diagnosis and treatment in underdeveloped parts of the world.

## Ethics Statement

Local Ethics Commitee: Hospital Universitário Walter Cantídio-Universidade Federal do Ceará.

We would like to outline that all patients provided written informed consent for the collection of samples and subsequent case report. All authors have read and agree with the manuscript publication. The local ethics committees approved the final version of this article.

## Author Contributions

PN and PB-N: conception and design of the work. PN, LM, FM, MK, CS, NM, MP, MS, and PB-N: acquisition, analysis, or interpretation of data for the work. PN, MP, MS, and PB-N: drafting the work. All authors were involved in critical revision of the manuscript for important intellectual content.

### Conflict of Interest Statement

The authors declare that the research was conducted in the absence of any commercial or financial relationships that could be construed as a potential conflict of interest.
